# Time-Lag Aware Latent Variable Model for Prediction of Important Scenes Using Baseball Videos and Tweets

**DOI:** 10.3390/s22072465

**Published:** 2022-03-23

**Authors:** Kaito Hirasawa, Keisuke Maeda, Takahiro Ogawa, Miki Haseyama

**Affiliations:** 1 Graduate School of Information Science and Technology, Hokkaido University, N-14, W-9, Kita-ku, Sapporo 060-0814, Japan; 2 Office of Institutional Research, Hokkaido University, N-8, W-5, Kita-ku, Sapporo 060-0808, Japan; maeda@lmd.ist.hokudai.ac.jp; 3 Faculty of Information Science and Technology, Hokkaido University, N-14, W-9, Kita-ku, Sapporo 060-0814, Japan; ogawa@lmd.ist.hokudai.ac.jp (T.O.); miki@ist.hokudai.ac.jp (M.H.)

**Keywords:** latent variable model, prediction of important scenes, Twitter, sports video, time lags

## Abstract

In this study, a novel prediction method for predicting important scenes in baseball videos using a time-lag aware latent variable model (Tl-LVM) is proposed. Tl-LVM adopts a multimodal variational autoencoder using tweets and videos as the latent variable model. It calculates the latent features from these tweets and videos and predicts important scenes using these latent features. Since time lags exist between posted tweets and events, Tl-LVM introduces the loss considering time lags by correlating the feature into the loss function of the multimodal variational autoencoder. Furthermore, Tl-LVM can train the encoder, decoder, and important scene predictor, simultaneously, using this loss function. This is the novelty of Tl-LVM, and this work is the first end-to-end prediction model of important scenes that considers time lags to the best of our knowledge. It is the contribution of Tl-LVM to realize high-quality prediction using latent features that consider time lags between tweets and multiple corresponding previous events. Experimental results using actual tweets and baseball videos show the effectiveness of Tl-LVM.

## 1. Introduction

The growth of various devices and network technologies has made several video distribution services available. Since sports videos attract many people, their distribution services, such as Rakuten Sports (https://sports.rakuten.com/, accessed on 1 February 2022) and DAZN (https://www.dazn.com/, accessed on 1 February 2022) provide many sports games. Although users accessing videos that they seek has become easy, real-time access to all games provided by these distribution services is difficult. Baseball, in particular, has a longer game time than other famous sports. Specifically, one soccer or basketball game lasts about two hours, while one baseball game lasts about three hours, that is, the length of the game time of the baseball game is 1.5 times longer than a soccer or basketball game. Thus, techniques for efficiently watching baseball games are required [1,2].

The generation of highlights from several important scenes, such as the run batted in (RBI), hit, and home run is widely known as a solution to assist efficient watching. To generate highlights, researchers have conducted various studies on detecting important scenes in sports videos [3,4,5,6]. However, since the editor generates the highlights by manually selecting these important scenes, it is difficult to assist viewers with efficient watching in real time. Alternatively, viewers can watch important scenes in real time by understanding when they will occur. Various studies have been conducted on methods for predicting these scenes [7,8,9]. The video-based prediction methods [7,8] use the game situation and visual appearance of the events in games. In these methods, situations such as the excitement of audiences and the scoring of games are captured by an effective representation of visual information. On the other hand, [9] showed that using both videos of e-sports games and audience chat reactions led to improved performance in predicting important scenes. Thus, using both visual information and audience reactions is effective for prediction. The use of audience reactions is expected to further improve the performance of methods using sports videos. Therefore, it is necessary to develop a new method to understand the reactions of viewers in sports videos.

Game viewers often post their messages, including their reactions, through microblogging services, and these messages explain the details of these games. Furthermore, Twitter (https://twitter.com/, accessed on 1 February 2022) is one of the most effective microblogging services for collecting information on the situation of games and viewers’ reactions. Twitter allows users to receive and post short text messages called *tweets*. Thus, using features extracted from both videos and tweets posted by viewers has improved the prediction and detection performance of important scenes in sports videos [10,11,12,13,14,15,16,17].

Several different modalities must be considered when using both tweets and videos. To consider these modalities, many researchers have proposed several methods that focus on the relationship among these modalities, such as tweets and visual and audio modes [18,19,20,21,22]. The video-text retrieval method [18] learns two joint spaces between multimodal features. It [19] learns multimodal embeddings across different modalities using deep canonical correlation analysis [23]. The method in [20] proposes a convolutional neural network-based medical image fusion algorithm to make the medical diagnosis more reliable and accurate. By obtaining medical images from different modalities, complementary as well as redundant information can be obtained. Furthermore, by combining the images from the camera and active sensor information, the method in [21] demonstrates how it outperforms a single modality in end-to-end artificial driving. The method in [22] uses a multimodal variational autoencoder (MVAE) [24] using tweets and visual information as the latent variable model (LVM) to detect fake news. The effectiveness of using LVM to consider both videos and tweets has also been reported in [14,16]. The MVAE can discover correlations between modalities by learning shared representations. Thus, it is expected that MVAE is effective in predicting important scenes in baseball videos using both tweets and videos. For constructing MVAE-based methods using tweets and videos, the following problem is considered: viewers watching baseball videos post tweets inspired by several previous events, such as an RBI hit and home run. Thus, since tweets and multiple corresponding events have time lags, the conventional methods [14,16] detect important scenes through the consideration of tweets and events. Specifically, the conventional method [14] detects important scenes via a time-lag-aware multimodal variational autoencoder that has the encoder considering the time lags between tweets and events, and the conventional method [16] detects them based on a generative adversarial network-based approach using features transformed via bidirectional time-lag aware deep multiset canonical correlation analysis. However, these methods cannot simultaneously train a time-lag aware feature transformation network and network for the detection of important scenes. By constructing an end-to-end LVM-based approach with consideration of the relationships between baseball videos and tweets, an accurate prediction method of important scenes can be expected.

In this paper, a time-lag aware LVM (Tl-LVM) for the prediction of important scenes in baseball videos is proposed. Tl-LVM adopts MVAE using tweets and videos as the LVM and achieves prediction using tweets and visual and audio features extracted from them. It comprises the encoder, decoder, and important scene predictor. The encoder converts multimodal features into latent features using neural networks that consider the time lags. In the decoder, the original features, which are inputs of the encoder, are recovered from the latent features using neural networks that are the inverse structure of the encoder. Moreover, the important scene predictor outputs the probability that the scene to be predicted is important from the latent features.

Novelty: In Tl-LVM, the encoder, decoder, and important scene predictor are trained simultaneously by minimizing the loss function with the loss based on the feature correlation as the new introduction into the loss function of the MVAE.

Contribution: Tl-LVM can achieve high-quality prediction using latent features that consider time lags between tweets and multiple corresponding previous events.

The remainder of the paper is organized as follows. In Section 2.1 and Section 2.2, the structure and loss function of Tl-LVM are described, respectively. Experimental results are described in Section 3 to verify the effectiveness of Tl-LVM. Moreover, Section 4 presents the discussion with an example of an important scene correctly predicted using Tl-LVM and the results when parameters are changed. Finally, Section 5 presents the conclusions.

## 2. Materials and Methods

**Figure 1:**
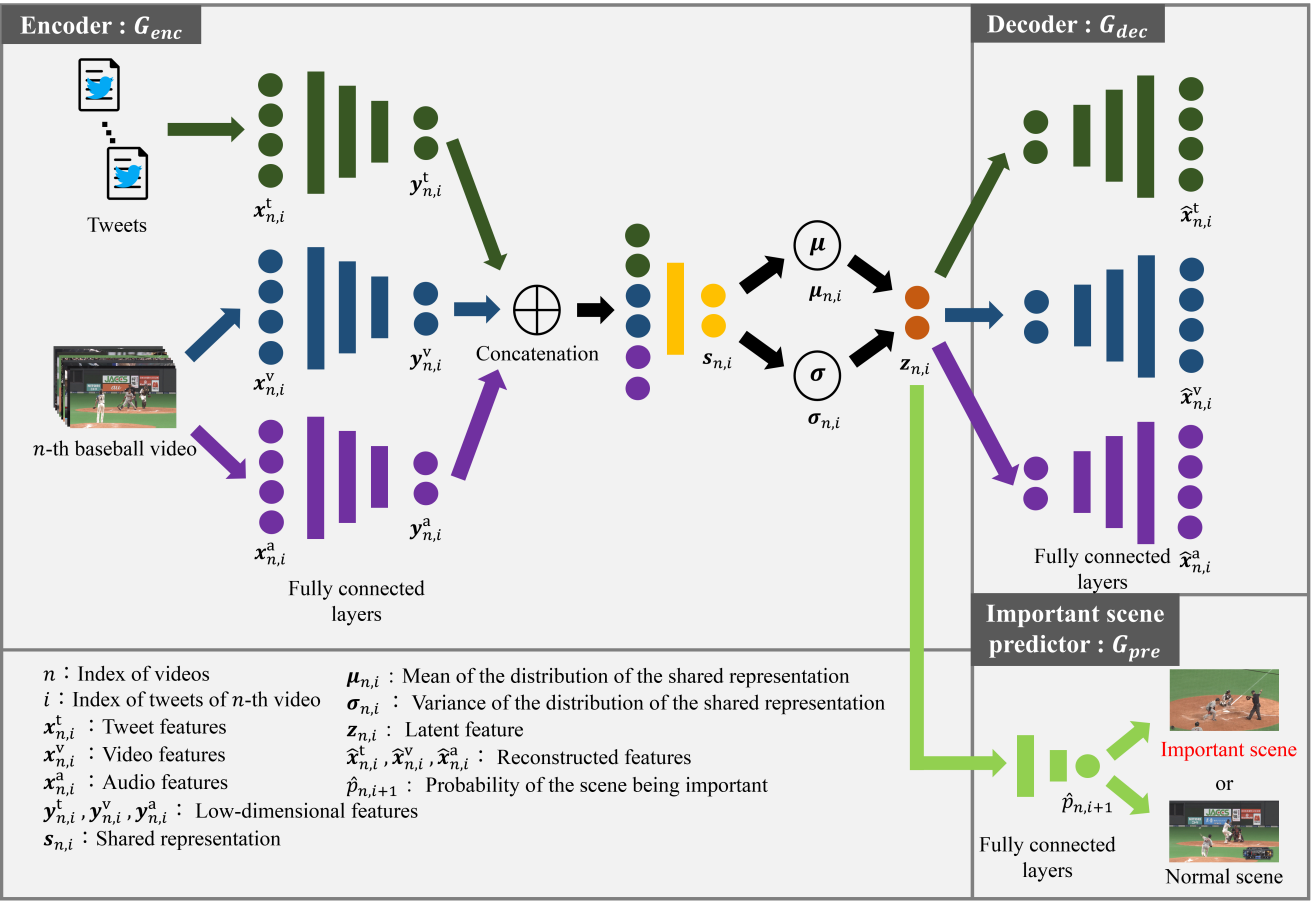
Overview of Tl-LVM. The encoder extracted latent features from features of tweets and baseball videos. By the decoder, original features were reconstructed from latent features. Additionally, the important scene predictor output the probability of the target scene being important and determined whether it was important or normal using the output probability. The encoder, decoder, and important scene predictor consisted of multiple fully connected layers, respectively. Unlike the conventional method [14] which trains a part of the encoder considering time lags, Tl-LVM can train the entire network considering time lags based on the new loss function. Therefore, this figure does not represent a time-lag aware transformation in the encoder as in the figure of the conventional method. While Tl-LVM is a model for prediction, the conventional method is a model for detection. Thus, Tl-LVM has the predictor network, but the conventional method has the detector network.

The overview of Tl-LVM is shown in Figure 1. To implement Tl-LVM, Python 3.6.9, keras 2.3.1, and tensorflow 1.14.0 were used on a computer with Ubuntu 18.04 LTS installed. The computer code used is not publicly available.

### 2.1. Network Structure

The input in the encoder was tweets and baseball videos, and latent features considering time lags between tweets and videos were the output. The encoder had three fully connected layers for tweets, visual features, and audio features as well as a fully connected layer inputting the features where they were concatenated. First, in a feature extraction architecture, Tl-LVM extracted features from tweets and baseball videos. Specifically, Tl-LVM extracted features $x_{n,i}^{m}\in R^{d_{x}}(i=1,2,\ldots ,I_{n};I_{n}$, which was the total tweets of *n*-th video) from *n*-th baseball video (n=1,2,…,N; *N* is the total videos) and corresponding tweets. Note that m∈{t,v,a} denotes the modality, such as tweet, visual, and audio modality. Consequently, Tl-LVM obtained feature matrices $X_{n}^{m}=\left[x_{n,1}^{m},\ldots ,x_{n,i}^{m},\ldots ,x_{n,I_{n}}^{m}\right]\in R^{d_{x}\times I_{n}}$. The details of the feature extraction architectures are explained in Section 3.1. Tl-LVM obtained low-dimensional features $y_{n,i}^{m}$ by passing $x_{n,i}^{m}$ through the three fully connected layers. By passing features concatenating these features $y_{n,i}^{t}$, $y_{n,i}^{v}$ and $y_{n,i}^{a}$ through a fully connected layer, Tl-LVM obtained the shared representation $s_{n,i}$. From this shared representation $s_{n,i}$, the mean $\mu_{n,i}$ and variance $\sigma_{n,i}$ of $s_{n,i}$ were obtained. Then, the latent features $z_{n,i}=\mu_{n,i}+\sigma_{n,i}\circ \epsilon$ were defined, where random variables ϵ were sampled from the Gaussian distribution. Note that $\sigma_{n,i}\circ \epsilon$ is the Hadamard product between $\sigma_{n,i}$ and ϵ. By denoting the encoder as $G_{\mathrm{enc}}\left(x_{n,i}^{m},\theta_{\mathrm{enc}}\right)$, latent features $z_{n,i}\in R^{d_{z}}$ are defined as

(1) $$z_{n,i}=G_{\mathrm{enc}}\left(x_{n,i}^{m},\theta_{\mathrm{enc}}\right).$$

Note that $\theta_{\mathrm{enc}}$ denote all parameters of the encoder.

The decoder is the network for reconstructing original features $x_{n,i}^{m}$ from latent features $z_{n,i}$. It has three fully connected layers for tweets, visual features, and audio features. Specifically, passing latent features $z_{n,i}$ through a fully connected layer makes the decoder output reconstructed features ${\hat{x}}_{n,i}^{m}$, defined as:

(2) $${\hat{x}}_{n,i}^{m}=G_{\mathrm{dec}}\left(z_{n,i},\theta_{\mathrm{dec}}\right).$$

Note that $G_{\mathrm{dec}}\left(z_{n,i},\theta_{\mathrm{dec}}\right)$ denotes the decoder, and $\theta_{\mathrm{dec}}$ denotes all parameters of the decoder.

The important scene predictor predicts whether the target scene is important or normal. It comprises two fully connected layers. The inputs are latent features $z_{n,i}$, and the output is the probability ${\hat{p}}_{n,i+1}$ of the scene corresponding to $z_{n,i+1}$ being important. The probability ${\hat{p}}_{n,i+1}$ is defined as:

(3) $${\hat{p}}_{n,i+1}=G_{\mathrm{pre}}\left(z_{n,i},\theta_{\mathrm{pre}}\right),$$

where the important scene predictor is denoted as $G_{\mathrm{pre}}\left(z_{n,i},\theta_{\mathrm{pre}}\right)$. Note that $\theta_{\mathrm{pre}}$ denotes all parameters of $G_{\mathrm{pre}}\left(z_{n,i},\theta_{\mathrm{pre}}\right)$. When ${\hat{p}}_{n,i+1}>\tau$, the (i+1)-th scene is predicted as an important scene, with a predetermined threshold value τ.

### 2.2. Loss Function

The loss function based on the feature correlation was newly introduced into MVAE. The parameters of the encoder, decoder, and important scene predictor were simultaneously optimized by the minimization of the loss function defined as:

(4) $$L_{\mathrm{final}}\left(\theta_{\mathrm{enc}},\theta_{\mathrm{dec}},\theta_{\mathrm{pre}}\right)=(\sum_{m\in \{t,v,a\}}\psi_{\mathrm{rec}}^{m}L_{\mathrm{rec}}^{m})+\psi_{\mathrm{kl}}L_{\mathrm{kl}}+\psi_{\mathrm{pre}}L_{\mathrm{pre}}+\psi_{\mathrm{cor}}L_{\mathrm{cor}},$$

where $L_{\mathrm{rec}}^{m}$ is the reconstruction loss. By training Tl-LVM to bring the output and imput of the decoder and encoder closer, respectively, latent features could be extracted to reconstruct features. Then, the probability distribution parameters (μ and σ) that closely resemble those of Gaussian distribution were optimized by minimizing the KL divergence $L_{\mathrm{kl}}$. Furthermore, $L_{\mathrm{pre}}$ is the prediction loss for important scenes. To balance individual terms of the loss function $L_{\mathrm{final}}$, Tl-LVM adopted parameters $\psi_{\mathrm{rec}}^{m}$, $\psi_{\mathrm{kl}}$, $\psi_{\mathrm{pre}}$, and $\psi_{\mathrm{cor}}$. Furthermore, Tl-LVM could be used to calculate these optimal parameters based on Adam [25] by minimizing $L_{\mathrm{final}}$.

Specifically, the reconstruction loss $L_{\mathrm{rec}}^{m}$ is defined as:

(5) $$L_{\mathrm{rec}}^{m}=\frac{1}{N}\sum_{n=1}^{N}\{\frac{1}{\left(I_{n}-1\right)d_{x}}\sum_{i=1}^{I_{n}-1}\sum_{d=1}^{d_{x}}{\left({\hat{x}}_{n,i,d}^{m}-x_{n,i,d}^{m}\right)}^{2}\}.$$

Note that ${\hat{x}}_{n,i,d}^{m}$ and $x_{n,i,d}^{m}$ are the *d*-th values of ${\hat{x}}_{n,i}^{m}$ and $x_{n,i}^{m}$, respectively.

The KL divergence $L_{\mathrm{kl}}$ is defined as:

(6) $$L_{\mathrm{kl}}=\frac{1}{N}\sum_{n=1}^{N}\{\frac{1}{2(I_{n}-1)}\sum_{i=1}^{I_{n}-1}\sum_{d=1}^{d_{z}}\left(\mu_{n,i,d}^{2}+\sigma_{n,i,d}^{2}-\log\left(\sigma_{n,i,d}\right)-1\right)\}.$$

Note that $\mu_{n,i,d}$ and $\sigma_{n,i,d}$ are the *d*-th values of $\mu_{n,i}$ and $\sigma_{n,i}$, respectively.

**Figure 2:**
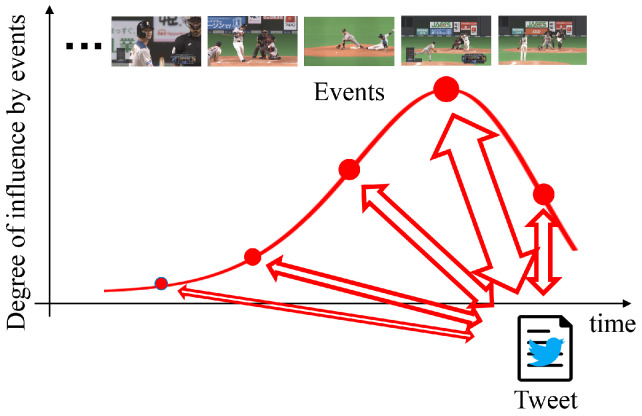
Relationship between multiple events and a tweet affected by them. Additionally, these events were weighted by the degree of influence defined using the Poisson distribution. This figure is an adaptation from Reference [14].

For constraining the values between zero and one, and representing approximations between ground truth and predictive labels, the prediction loss of important scenes $L_{\mathrm{pre}}$ was defined as:

(7) $$L_{\mathrm{pre}}=-\frac{1}{N}\sum_{n=1}^{N}\{\frac{1}{I_{n}-1}\sum_{i=2}^{I_{n}}\left(l_{n,i}\log\left({\hat{l}}_{n,i}\right)+\left(1-l_{n,i}\right)\log\left(1-{\hat{l}}_{n,i}\right)\right)\},$$

where ${\hat{l}}_{n,i}$ is the predicted label calculated using the probability ${\hat{p}}_{n,i}$ output from the important scene predictor, and $l_{n,i}$ is the ground truth label. Moreover, for the time-lag aware training, the loss function based on the feature correlation was introduced. Specifically, tweets are posted by viewers influenced by multiple events, not just a single event during the baseball game. Thus, there are correlations between tweet features and visual and audio features of several events corresponding to this tweet. Since viewers post tweets after events occur, these posted tweets are strongly influenced by the immediately preceding event and gradually become weaker. From this disposition, Tl-LVM assumes that tweets correspond to events from present to past in response to the degree of influence using the Poisson distribution, as shown in Figure 2. From this assumption, $L_{\mathrm{cor}}$ was defined in [26] as:

(8) $$L_{\mathrm{cor}}=-\frac{1}{d_{x}\left(M-1\right)}\sum_{k=1}^{d_{x}}\frac{\phi_{k}^{⊤}C_{B}\phi_{k}}{\phi_{k}^{⊤}C_{W}\phi_{k}},$$

where $\phi_{k}\in R^{d_{x}}\left(k=1,2,\ldots ,d_{x}\right)$ denotes the optimal weight common to all modalities. Note that *M* is the number of modalities. The matrix $C_{B}$, which is the between-set covariance matrix considering the time lags, and $C_{W}$, which is the within-set covariance matrix, were defined as:

(9) $$\begin{array}{cc} C_{B} & =\sum_{n=1}^{N}\sum_{m_{1}\in \left\{t,v,a\right\}}\sum_{m_{2}\in \left\{t,v,a\right\},m_{2}\neq m_{1}}{\bar{R}}_{n}^{m_{1},m_{2}}, \end{array}$$

(10) $$\begin{array}{cc} C_{W} & =\sum_{n=1}^{N}\sum_{m\in \{t,v,a\}}{\underline{R}}_{n}^{m,m}. \end{array}$$

Note that the above equations omit the same scaling value ${\left(I_{n}-1\right)}^{-1}M^{-1}$. ${\bar{R}}_{n}^{m_{1},m_{2}}$ and ${\underline{R}}_{n}^{m,m}$ were defined as:

(11) $${\bar{R}}_{n}^{m_{1},m_{2}}=\left\{\begin{array}{cc} \; & \frac{\sum_{l=0}^{L-1}\frac{e^{-\lambda }\lambda^{l}}{l!}{\tilde{X}}_{n,0}^{m_{1}}{\tilde{X}}_{n,l}^{m_{2}⊤}}{\sum_{l=0}^{L-1}\frac{e^{-\lambda }\lambda^{l}}{l!}}\\ \left(m_{1}\in \left\{t\right\}\cap m_{2}\in \left\{v,a\right\}\right)\\ \; & \frac{\sum_{l=0}^{L-1}\frac{e^{-\lambda }\lambda^{l}}{l!}{\tilde{X}}_{n,l}^{m_{1}}{\tilde{X}}_{n,0}^{m_{2}⊤}}{\sum_{l=0}^{L-1}\frac{e^{-\lambda }\lambda^{l}}{l!}}\\ \left(m_{1}\in \left\{v,a\right\}\cap m_{2}\in \left\{t\right\}\right)\\ \; & {\tilde{X}}_{n,0}^{m_{1}}{\tilde{X}}_{n,0}^{m_{2}⊤}\;\left(\mathrm{otherwise}\right) \end{array}\right.,$$

(12) $${\underline{R}}_{n}^{m,m}={\tilde{X}}_{n,0}^{m}{\tilde{X}}_{n,0}^{m⊤},$$

where λ controls the peak of the Poisson distribution, and *L* is the impact range of events correlating with the target tweet. Note that λ equals the variance and mean of the distribution. ${\tilde{X}}_{n,l}^{m}=\left[X_{n,L-l}^{m},\ldots ,X_{n,I_{n}-1-l}^{m}\right]\;\left(l=0,\ldots ,L-1\right)$ are mean-normalized feature matrices.

For training with the consideration of time lags, the loss function based on the feature correlation was newly introduced into the loss function of MVAE from Equation (4). This way, Tl-LVM achieved simultaneous training of the encoder, decoder, and important scene predictor by considering time lags. This is the novelty of this paper. The original MVAE uses simple encoding and decoding networks, meaning that these networks cannot consider time lags between different modalities. However, Tl-LVM focuses on the relationships between tweets and visual and audio features and is flexible enough to capture these time lags from Equation (11). With the above novelty, Tl-LVM achieved a more accurate prediction.

## 3. Results

### 3.1. Experimental Setting

An experiment was conducted to verify the effectiveness of Tl-LVM. Although public datasets are generally used in computer vision, private datasets generated by the authors in experiments using videos or tweets are often used [17]. Therefore, since the novelty of Tl-LVM cannot be verified by public datasets, a private dataset was adopted. As the private dataset,12 baseball videos broadcast on Pacific League TV from 14 June to 27 September in 2019, and tweets posted during these games, were collected. These tweets were collected using the query “#lovefighters”, which is an official hashtag of the baseball team. Note that these videos are 30 frames per second, and details of these videos are shown in Table 1. Seven randomly-selected games out of the 12 games were used as training data, and the other five games were used as test data. Since ten and six games, respectively, were used in previous sports video analysis experiments [4,27], 12 games were considered sufficient for this experiment. To extract tweet features from tweets, Tweet2Vec [28], which extracts strong features for Twitter-specific abbreviations, typos, and slang, was used. Tweet2Vec has a bi-directional gated recurrent unit [29], a softmax layer, and a linear layer. For training Tweet2Vec, tweets were collected using 27 hashtags associated with professional baseball. 3D ResNet [30] was employed to extract visual features. This 3D ResNet was pre-trained using the Kinetics dataset [31], which is a large-scale dataset consisting of various human behavior. Three-dimensional ResNet consists of a softmax layer, a global average pooling layer, a fully connected layer, and 17 convolutional blocks. Furthermore, VGG16, which consists of five max layers, three fully connected layers, a softmax layer, and 13 convolution layers, was used to extract audio features. VGG16 was pre-trained on the ImageNet dataset [32]. Although it is typically used to extract features from images, spectrogram-based feature computation with a pre-trained CNN model can be effective in representing audio data [33,34]. Thus, Tl-LVM applied the pre-trained VGG16 to extract audio features from spectrograms. In addition, $d_{X}$, $d_{z}$, τ, $\psi_{\mathrm{rec}}^{m}$, $\psi_{\mathrm{kl}}$, $\psi_{\mathrm{isp}}$, *L*, and λ were empirically set to 500, 64, 0.5, 1, 1, 1, 12, and 3, respectively. Furthermore, by conducting experiments with several values for each parameter, $d_{X}$, $d_{z}$, τ, *L*, and λ were set to values at which Tl-LVM had the highest f-measure. Additionally, $\psi_{\mathrm{rec}}^{m}$, $\psi_{\mathrm{kl}}$, and $\psi_{\mathrm{isp}}$ were set to one according to [22].

To confirm the validity of Tl-LVM, the following comparative methods (Comps. 1–11) were used. The following Comps. 1–7 were employed to ensure effective flexible representation of tweets and visual and audio features. To verify the effectiveness of considering the time lags between tweets and videos, the following Comp. 8 was adopted. Then, Comps. 9 and 10 were adopted to compare MVAE and other models for the prediction. Additionally, Comp. 11 was adopted to confirm the effectiveness of the end-to-end prediction model.

Comps. 1–6: This considers methods adopting features according to Table 2. Since there are time lags between “tweet features” and “visual and audio features”, Comps. 1–3 and 6 do not consider time lags.

Comp. 7: A method simply integrating prediction results constructed for each modality. This method estimates important scenes using the majority vote of prediction results of Comps. 1–3.

Comp. 8: A method not considering time lags according to MVAE [22].

Comp. 9: A method inputting features transformed using the deep multiset canonical correlation analysis [35], which can flexibly express relationships across heterogeneous features, into MVAE.

Comps. 10: A method that predicts important scenes using a long short-term memory [36], which is effective for the relationships between time series data. Similar to Tl-LVM, videos and tweets are input, and the probabilities of the scene being important are output.

Comps. 11: A method from the previous method [16]. This method separately trains the network for predicting important scenes and for the feature transformation considering time lags.

Ground truth given by eight subjects with 11–15 years of baseball experience was used. Note that these subjects are healthy males between the ages of 20 and 24. They gave the label that the target scene will be important or normal. Then, the performance of Tl-LVM and Comps. were evaluated using F-measure.

### 3.2. Performance Evaluation

The F-measures of Tl-LVM and Comps. 1–11 are shown in Table 3. The effectiveness of Tl-LVM can be observed by comparing its respective f-measures. Specifically, since the F-measures of Tl-LVM are higher than those of Comps. 1–6, it is confirmed that the use of tweets and visual and audio features is more effective for prediction. Furthermore, from the results of the comparison between Tl-LVM and Comp. 7, it is asserted that simply adopting these features does not necessarily have a positive effect on performance improvement. Therefore, it is verified that considering the relationship between videos and tweets is effective for prediction. Furthermore, f-measure of Tl-LVM is higher than that of Comp. 8, and thus, it is clarified that considering time lags between “tweet features” and “visual and audio features” is effective. Although Comps. 9 and 10 can flexibly capture the relationship among multimodal features, the f-measures of these methods are lower than that of Tl-LVM. Therefore, the MVAE-based approach is effective for predictions dealing with tweets and videos. Additionally, from results of Tl-LVM and Comp. 11, it is verified that training all networks in an end-to-end manner is effective. Since predicting important scenes is very difficult, it is not easy to achieve a high F-measure. Therefore, in order to evaluate the superiority of Tl-LVM, an additional evaluation index is adopted. Specifically, specificity when sensitivity was almost 1.0 (i.e., maximizing sensitivity) was adopted. Specificity when sensitivity is almost 1.0 means how much over-prediction of normal scenes can be reduced when accurately predicting almost every important scene of the ground truth. Specificity in Tl-LVM and Comp. 11, when sensitivity is almost 1.0, was 0.392 and 0.376, respectively. This result means that it can suppress the over-prediction of normal scenes by 1.6% more than Comp. 11, even if almost all important scenes in the ground truth are accurately predicted. Consequently, an accurate prediction using Tl-LVM can be realized.

## 4. Discussion

**Figure 3:**
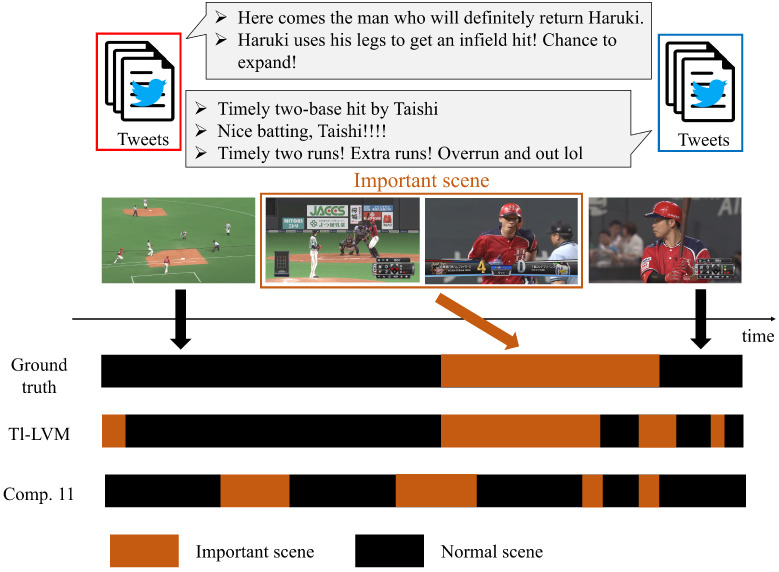
Example of an important scene correctly predicted by Tl-LVM and tweets containing content from this scene. Furthermore, the horizontal axis represents time. Ground truth corresponding to this scene and labels (predicted by Tl-LVM and Comp. 11) are shown with respect to time. There is a time lag between when this important scene occurs and when these tweets are posted.

An example of an important scene correctly predicted using Tl-LVM, tweets containing content from this scene, ground truth corresponding to this scene, and labels (predicted by Tl-LVM and Comp. 11) are shown in Figure 3. Specifically, the important scene surrounded by brown rectangles and in which the player has an RBI double is the one predicted by Tl-LVM. Interestingly, the viewer’s expectations for this scene are contained in the body of the tweet surrounded by the red rectangle. Since the viewers posted their tweets before this scene occurred, using these tweets for prediction was shown to be effective. However, the body of tweets surrounded by the blue rectangle denotes the results and enjoyment of viewers watching this scene. Therefore, it can be seen that the important scene influences these tweets. Furthermore, from this Figure 3, Tl-LVM predicts important scenes more accurately than Comp. 11, which separately trains the network for prediction and feature transformation considering time lags, respectively. Consequently, it can be confirmed that Tl-LVM, which simultaneously trains the encoder, decoder, and important scene predictor, accurately predicts important scenes by considering tweets and videos.

Table 4 shows the average F-measure of Tl-LVM for all games when parameters λ and *L* of the Poisson distribution are changed. Since the results when parameters λ are changed suggest how much the peak of the distribution should slide, how much time lag exists between the event and posted tweet can be calculated. Moreover, the results when parameters *L* are changed show how events influence the tweet. From Table 4, it can be seen that the highest F-measure is obtained where λ and *L* are set to 3 and 12. Then, since viewers post the test data tweet on average every 24 s, this graph shows that the time lag between an event and its associated tweet is about 72 s and that amounts to 288 s of affect tweets. From the above discussion, it was confirmed that the calculation based on the parameters λ and *L* is effective for revealing the correlations between tweets and videos. By utilizing these parameters, high-quality prediction using the LVM approach that considers time lags between tweets and events can be realized.

## 5. Conclusions

In this paper, Tl-LVM using baseball videos and tweets for predicting important scenes was proposed. Tl-LVM adopted MVAE, which can discover correlations across modalities, as the latent variable model. It calculates the latent features from these tweets and videos and predicts important scenes using these latent features. By introducing the loss function considering time lags based on the feature correlation into the loss function of the MVAE, Tl-LVM can be used to train the encoder, decoder, and important scene predictor, simultaneously. To the best of our knowledge, Tl-LVM is the first end-to-end prediction model of important scenes that considers time lags between tweets and videos. With this novelty, Tl-LVM has the contribution of being able to achieve high-quality prediction using latent features that consider time lags between tweets and multiple corresponding previous events. Furthermore, the effectiveness of Tl-LVM was verified using experimental results. In future work, the parameter of a Poisson distribution λ and *L* will be automatically determined using each characteristic of the baseball events. Therefore, the loss function, which flexibly considers these differences in the parameter, will be constructed. 

## Figures and Tables

**Table 1:** Details of the baseball videos used in the experiment.

Games	Game Time	Number of Important Scenes	Average Time Length of Important Scenes
1	3 h 23 min	12	2 min 28 s
2	2 h 59 min	14	2 min 31 s
3	3 h 32 min	22	2 min 03 s
4	3 h 08 min	18	1 min 58 s
5	2 h 44 min	11	1 min 40 s

**Table 2:** Features used in Comps. 1–6.

Features	Comp. 1	Comp. 2	Comp. 3	Comp. 4	Comp. 5	Comp. 6
Tweet	✓			✓	✓	
Video		✓		✓		✓
Audio			✓		✓	✓

**Table 3:** F-measure of important scene prediction in Tl-LVM and Comps. 1–11. Bold values represent the highest F-measure.

Games	Tl-LVM	Comp. 1	Comp. 2	Comp. 3	Comp. 4	Comp. 5
1	**0.559**	0.469	0.457	0.456	0.502	0.481
2	**0.405**	0.355	0.366	0.334	0.385	0.378
3	**0.487**	0.402	0.412	0.404	0.408	0.412
4	**0.365**	0.330	0.325	0.323	0.345	0.345
5	**0.390**	0.334	0.314	0.298	0.334	0.314
Average	**0.441**	0.378	0.375	0.363	0.395	0.386
**Games**	**Comp. 6**	**Comp. 7**	**Comp. 8 [22]**	**Comp. 9 [35]**	**Comp. 10 [10]**	**Comp. 11 [16]**
1	0.527	0.440	0.502	0.517	0.525	0.552
2	0.378	0.385	0.393	0.385	0.387	0.400
3	0.408	0.394	0.451	0.456	0.465	**0.487**
4	0.344	0.345	0.357	0.352	0.353	0.360
5	0.306	0.358	0.364	0.364	0.357	0.381
Average	0.393	0.384	0.413	0.415	0.417	0.436

**Table 4:** Average F-measure of Tl-LVM for all games when parameters λ and *L* of the Poisson distribution are changed. Bold values represent the highest F-measure.

	L=10	L=12	L=14	L=16	L=18	Average
λ=1	0.417	0.436	0.415	0.403	0.392	0.413
λ=3	0.436	**0.441**	0.431	0.417	0.403	0.426
λ=5	0.415	0.427	0.403	0.407	0.386	0.408
λ=7	0.403	0.409	0.403	0.392	0.378	0.397
λ=9	0.395	0.413	0.386	0.378	0.367	0.388
Average	0.413	0.425	0.408	0.399	0.385	0.406
